# Editor’s Book Table

**Published:** 1870-08

**Authors:** 


### Editor's Book Table.

*Fourth Annual Report of the Metropolitan Board of Health of the State of New York.* 1869. New York: D. Appleton & Company, 90, 92 and 94 Grand street. 1869. Pp. 564.

The present volume is, we should say, practically the most valuable one of the series indicated.

The Sanitary Board, under Superintendent Elisha Harris, M.D., assisted by Moreau Morris, M.D. and R. Cresson Stiles, M.D. together with a corps of ten medical inspectors, have accomplished an amount of work of which both they and the profession represented have reason to be proud. It is possible that New York City is, as the newspapers assert, badly governed, but the book before us proves that its Sanitary Board is worthy of the highest praise. A student of hygiene can scarcely find elsewhere a more complete resumè of what sanitary science demands and is capable of affording.

Some idea of the magnitude of their work may be gathered from the fact that the number of reports for the year were 28,321. This included, in addition to the ordinary routine of duties, special investigations having for their object the thorough reconstruction of tenement houses, the closure of uninhabitable cellars, the protection of the city against small pox, by systematic vaccination, the inspection of immigrant vessels, the suppression of the sale of dangerous kerosene, the analysis of the milks brought to market and sold to families, the examination of the flour and bread used by the poor, the analysis of the air of public halls, schools, churches, etc., etc., microscopic and other observations on the water in use, plans for flushing the streets and sewers with salt water, for regulating the manufacture of gas, for improved methods of ventilation, etc., etc.

The vital statistics are presented by Superintendent Harris in a very clear and satisfactory manner, and, by means of a folding leaf, a diagram presents the general result most impressively.

We wish books of this character were more generally sought after by the profession, even though the money they cost had to be taken from investments in "Materia Medicas" and badly tasting and smelling drugs. But the time is coming rapidly when the laws of life and health will command their appropriate study.

*Superstition and Force. Essays on the Wager of Law—the Wager of Battle—The Ordeal—Torture.* By HENRY C. LEA. Second Edition, revised. Philadelphia: Henry C. Lea. 1870. Pp. 480.

The name of Lea—father and son—has been familiar to all medical book readers of this and the preceding generation. The great Philadelphia publishing house, by sagacity and tact in the choice of material for publication, by liberal arrangements with authors and honorable dealings with the public, have commanded success as well as high business distinction. But, perhaps, it is not generally known, to the profession, that the present representative of the old house, Henry C. Lea, has by several of his own productions manifested a variety and exactness of acquirement, a scholarly culture and philosophical power, which have rarely been equaled and still more rarely surpassed by any American author.

The subjects discussed in the present work are sufficiently set forth in the title. In one sense they are not medical, but in a higher sense they fall within the physician's domain. All influences that sway human minds, co-operate with the physical influences that move the atoms of the body. The grandest sphere of the physician's art is that which comprehends his power of influencing the corporeal changes by the high powers of the mind. Even educated medical men are too apt to neglect this grandest therapeutic energy, leaving it to be prostituted to the base uses of pitiful exclusivists and charlatans. When the surly English physician advised the medical student to read Don Quixotte as the best means of preparing himself for practice, we are not so sure but he intended a useful lesson. We are *quite sure* that Don Quixotte describes certain peculiarities of insanity much more successfully than does a recent essay specially devoted to the topic.

It is unfortunate that Psychological Medicine, so called, is too often taught merely as a speculative abstraction, or else as a means of conveyance of the grossest materialism tricked out in scientific phraseology. The best study of Psychology is afforded in the careful perusal of the works of painstaking, conscientious and impartial chroniclers of the operations of the human mind in times gone by, direct observation of mental actions now, both by personal association and reading, and then correction of all judgment by common sense. The practitioner or student who thinks he finds all of medical science in the technical anatomies, physiologies, materia medicas, or even cyclopædic works on "principles and practice," is as much at fault as one who expects to master metaphysics and the moral law by the scalpel and microscope. Physical forces correlate, but they correlate in a proportion expressed by numbers. Who will give us the numeral equivalents for Hamlet or—Dan. McFarland?

But, *revenons a nos moutons*—we know of no single work which contains, in so small a compass, so much illustrative of the strangest operations of the human mind. Foot notes give the authority for each statement, showing vast research and wonderful industry. We advise our confreres to read this book and ponder its teachings.

[NOTE.—All works reviewed in the columns of the CHICAGO MEDICAL JOURNAL may be found in the extensive stock of W. B. KEEN & COOKE, whose catalogue of Medical Books will be sent to any address upon request.

---

### Pamphlets.

*As regards Protoplasm, in relation to Professor Huxley's Essay on the Physical Basis of Life.* By JAMES HUTCHINSON STIRLING, F. R. C. S., LL.D., Edin. New Haven, Conn.: Charles C. Chatfield & Co. 1870. Pp. 71. For sale by S. C. Griggs & Co., Chicago. Price, 25 cents.

This is No. 3 of the University Series, the two former of which have been noticed (*vide* p. 230). It is one of the best of the numerous answers to Prof. Huxley's "premature generalizations." The publishers of this cheap series of valuable lectures and essays are conferring a great favor on the reading public. We notice that they are receiving numerous orders in advance of the publication or even announcement of forthcoming papers. This confidence is fully merited.

*The Bromides; their Physiological Effects and Therapeutical Uses.* By Z. C. MCELROY, M.D., Zanesville, Ohio, President of the Muskingum County Medical Society. [Reprinted from the New York Med. Jour., July, 1870.] New York: D. Appleton & Co. 1870. Pp. 26.

This paper, characterized by the careful study and close thought of its author, well known to our readers, concludes with the following propositions, which, aside from the evidences he presents, from our personal observations we are prepared fully to indorse:

1. That, from the inherent relations of bromine, and the bromides, to the organic tissues and structures of the human body, their physiological and therapeutical effects must always be that of promoting destructive metamorphosis, or waste: first, of all matter below the normal dynamic condition; second, of tissue or structure of type or form foreign to the human body; and, lastly, of the normal tissues themselves.

2. That they are never indicated, therapeutically, except when there is matter or tissue in the body which it is desirable to eliminate from it.

3. That they possess neither inherited nor acquired anæsthetic properties, nor hypnotic effects, as chloroform, ether, opium, or cannabis indica, which act by retarding destructive metamorphosis; but may, by promoting destructive metamorphosis of retained effete matter, be followed indirectly by anæsthetic or hypnotic effects, in the same way as the evacuation of the bladder of retained urine by the catheter.

4. That they are contra-indicated where nutrition is much impaired, or the rate of tissue-waste more than natural, and where structural changes, or loss of form, by substituting tissue of lower for that of higher organization, have impaired the dynamic integrity of the nerve-masses, as in locomotor ataxy, insanity, spinal paresis, etc.

5. That they are only indicated where the organic processes of life are restrained or interfered with, by adventitious circumstances, to resume their normal working on the removal of the restraint.

6. That their effects on persons living luxuriously, and leading inactive or sedentary lives, and on all in whom tissue-metamorphosis is sluggishly performed, is to increase the rate of waste, and to compensate, to some extent, for the physical exercise necessary to maintain the normal velocity of tissue-waste or changes.

*The Treatment of Croup.* By FORDYCE BARKER, M.D., Clin. Prof. of Midwifery and Dis. of Women in Bellevue Hospital, etc., etc. Reprinted from Am. Jour. of Obstetrics and Dis. of Women and Children; with remarks by one of the Editors, A. JACOBI, M.D., Clin. Prof. of Dis. of Children in Coll. of Phys. and Surgeons, N. Y. New York: W. A. Townshend & Adams, Publishers.

The names of Professors Barker and Jacobi are well known to the profession, and we wish that both they and those that may follow them in the publication of monographs, would forego cumbering title pages with voluminous explanations of the *status*. Recently in the JOURNAL we noticed Dr. Lente's positive assurance that pseudo-membranous croup was utterly shorn of its terrors by teaspoonful doses of calomel [SEDATIVE!]. But Professors Barker and Jacobi, singularly enough, deem it necessary to say something more—hence this pamphlet. Attention is especially called to the diagnosis. Prof. Barker objects to the term "false croup," and cannot give his unqualified assent to the "spasmodic laryngitis," recognized by Prof. Jacobi and most of the authorities. He thinks "the difference between false and true croup is essentially a question of intensity and extent of tissue involved, and there is no other radical difference between them," and that the terms false and true croup should be abandoned, as they convey a false impression. The croup of diphtheria he regards as an altogether distinct and specific disease.

Prof. B. relies almost wholly upon three or five grain doses, according to the age of the child, of turpeth mineral, repeated in fifteen minutes, unless vomiting occurs. So confident is he of its efficiency, that it is the only medicine which he has constantly carried in his pocket for twenty-eight years,-and he has no doubt that at this moment a hundred families in New York city have three-grain powders of the turpeth mineral, carefully labeled "croup powders." He thinks the powders do no harm, and if he finds on the morning visit that the case proves to be one of laryngismus stridulus, he has only to seek for the cause of the reflex irritation, and remove it in the usual manner. Subsequent febrile symptoms he controls by measures of which veratrum viride is the central figure. The treatment recommended by Prof. Barker, like that of Dr. Lente, has the advantage of definiteness and simplicity. It would be exceedingly gratifying if the results proved as favorable in the hands of other practitioners as in those of the leading advocates.

*The Third Annual Report of the Brainard Free Dispensary of the City of Chicago, for the year ending June* 30, 1870. The Dispensary is located at the north-west corner of Jefferson and Randolph streets, in Rice & Jackson's Building.

This institution is supported by voluntary contributions, and under the management of its efficient corps of managers, surgeons and physicians, is rapidly proving a great public charity. $40 paid at once, or $10 annually, constitutes the donor a life member. $5 gives an annual membership. All persons interested in the welfare of the sick poor are cordially invited to visit the dispensary, and witness its practical workings. During the year ending June 1st, 1870, 3,036 different persons were treated at the dispensary; 2,687 visits were made at the homes of the sick; 987 vaccinations were performed.

We are gratified to notice that the Board of Supervisors of Cook county, recognizing the beneficial influence of the dispensary, has appropriated $500 toward the current expenses of the year. The annual report contains a tabulated list of the diseases treated.

*Woman's Medical College of the New York Infirmary*, 128 *Second Avenue. Annual Catalogue and Announcement.*

This is a completely organized college, under the auspices of a large and influential board of trustees. The course of instruction as announced is complete and thorough, and the character of that instruction is guaranteed by the association of a board of examiners, consisting of Drs. Willard Parker, Isaac E. Taylor, Austin Flint, Stephen Smith, B. W. McReady, A. L. Loomis, and Samuel St. John.

If any of our fair friends are determined to undertake the essentially unfeminine business of practicing medicine, we most certainly recommend them first to thoroughly prepare themselves by attending this or some other as respectable college for women. They can get an announcement and all necessary information by addressing Dr. Emily Blackwell, Secretary of the Faculty, 128 Second Avenue, New York City.

Meanwhile, we warn them that the public do not want female practitioners who do not mingle some quackery with their craft. Astrology, in this city, takes better even than clairvoyance, and we have no doubt the same is true in New York.

*A Medico-Legal Study of the Case of Daniel McFarland.* By WILLIAM A. HAMMOND, M.D., Prof. Diseases of the Mind, etc., etc. Read before the Medico-Legal Society of the City of New York, May 12th, 1870. New York: D. Appleton & Company. Pp. 35.

A curious tractate, which the high professional respect we have always entertained for its author, forbids us from further noticing.

*New York State Inebriate Asylum. A Circular by the Superintendent*, D. G. DODGE, M.D., Binghampton, N. Y.

Containing principles of management, mode of gaining admission, rules for patients, etc. Copies can probably be obtained by addressing the superintendent as above.

*Steiger's Literarischer Monatsbericht.* (*Literary Monthly Record.*) May, 1870. Pp. 72. 12mo. New York: E. Steiger.

Steiger's Monatsbericht, commenced May, 1869, the only literary periodical published in the German language in the United States, enters upon its second volume, the first number of which has just been issued. Among its interesting contents will be found an article on the distinguished historian, Friedrich Kapp, who has just returned to Europe after twenty years' sojourn in this country, and whose works, some of which have been translated, are widely known,—an inquiry, "Which is the first German book printed in America?"—The "Poppenhusen Institute" in College Point, an institution generously founded and endowed by a German, for the advancement of knowledge and the improvement of the moral and social condition of the working classes,—an account of the "Growth of the Book and News Trade in the United States," with special reference to H. H. Bancroft & Co. and Sinclair Tousey,—besides many minor notes connected with schools and other matters of value to all literary men. We notice also the prospectus of a list to be compiled of all who have written German books and pamphlets in the United States; but the most striking feature of the present number of the Literary Monthly Record, in our opinion, as it will be in that of its numerous readers, is the announcement of a prize of eight hundred dollars offered for the best historical sketch of the intellectual vigor and progress of the German population in North America, more particularly exhibiting the influence of the German-American press on the development of American institutions. Such an announcement is calculated to awaken curiosity, and stimulate the exertions of many students and professional writers. Further particulars, we doubt not, will be eagerly sought after; and they can be found in the periodical itself, which may be obtained free by addressing Steiger's Literarischer Monatsbericht, 22 and 24 Frankfort Street, New York. It should be added here that the publisher continues to send his Monatsbericht free and prepaid to all who wish to have it.

*The Little Corporal Magazine.* Chicago: Sewell & Miller.

The July number of this beautiful juvenile comes to us greatly enlarged and improved, as well as finely illustrated. The wonderful growth of this young Napoleon of the juveniles has been as surprising as it is interesting. Its circulation has shot far ahead of that of any of its competitors. Its matter is entirely original and of a very high order. The freshness and vivacity of its pages cause the eyes of all our young people to sparkle. In its new, improved form it is one of the handsomest, as it is the cheapest, magazines we have ever seen. Childlike but not child*ish*, it rejoices the hearts of both parents and children alike. This number begins a new volume; now is a good time to subscribe. One dollar a year; sample copy 12 cents.

